# Comparison of efficiency of different self-assessment instruments for screening dysphonia

**DOI:** 10.1590/2317-1782/20232021123en

**Published:** 2023-04-17

**Authors:** Priscila Oliveira, Héryka Maria Oliveira Lima, Maiara dos Santos Sousa, Larissa Nadjara Almeida, Hêmmylly Farias da Silva, Ana Celiane Ugulino, Anna Alice Almeida, Leonardo Lopes

**Affiliations:** 1 Departamento de Fonoaudiologia, Centro de Ciências da Saúde, Universidade Federal da Paraíba - UFPB - João Pessoa (PB), Brasil.; 2 Departamento de Estatística, Universidade Federal da Paraíba - UFPB - João Pessoa (PB), Brasil.

**Keywords:** Voice, Dysphonia, Self-Testing, Mass Screening, Decision Making, Speech, Language and Hearing Sciences

## Abstract

**Purpose:**

To compare the efficiency of different vocal self-assessment instruments for dysphonia screening.

**Methods:**

262 dysphonic and non-dysphonic individuals participated in the research. The mean age was 41.3 (±14.5) years. The diagnosis of dysphonia was based on the auditory-perceptual analysis of the sustained vowel “é” and on laryngological diagnosis. The responses of the instruments were collected: Voice-Related Quality of Life (V-RQOL), Voice Handicap Index (VHI), VHI-10, Voice Symptoms Scale (VoiSS), and the Brazilian Dysphonia Screening Tool, (Br-DST) called in Brazilian Portuguese *Instrumento de Rastreio da Disfonia* (IRD^BR^). To analyze assertiveness in relation to the presence of dysphonia, the cutoff points of each instrument and the decision rule recommended by the IRD^BR^ were used. An exploratory analysis was performed to compare mean scores of instruments and verify associations between variables.

**Results:**

The instruments evaluated were sensitive to capture the impact of dysphonia in a similar way regardless of professional voice use and type of dysphonia. There was a difference only in VoiSS scores for the variable gender, with a higher score for females. Regarding global assertiveness, the instruments showed high rates of success in classification, with emphasis on the VoiSS, which had the highest rate (86.3%), followed by the IRD^BR^ (84.0%), VQL (80.9%), VHI (78.2%), and VHI-10 (75.2%).

**Conclusion:**

The VoiSS has the highest assertiveness index in the identification of dysphonia, followed by the IRD^BR^. The IRD^BR^ is a short, simple, and easy-to-apply tool for screening procedures.

## INTRODUCTION

Voice disorders affect 3 to 9% of the population and produce a series of negative impacts on the quality of life of individuals^(1,2)^. Therefore, the importance of a complete and efficient vocal evaluation is paramount for the diagnosis of a possible voice disorder, which includes auditory, acoustic, and aerodynamic perceptual analysis of voice, in addition to laryngeal examination and evaluation from the patient's perspective^(3)^.

However, performing a full-scale voice assessment procedure is not always feasible, as the diagnosis of a voice disorder is a process that demands time, material resources, and specialized professionals. In population surveys or preventive campaigns, specific mechanisms are recommended for screening, as they aim to select individuals with high chances of presenting dysphonia early in order to be referred for a complete confirmatory diagnostic evaluation at a later time^(4)^. Therefore, a screening tool does not need to be a complete diagnostic tool. The use of short screening tools for the pre-selection of at-risk individuals to be referred for later diagnostic confirmation may allow expanding the scope and resolution of preventive epidemiological actions in the field of voice^(5,6)^.

The indispensable requirements for a screening instrument are easy application, broad usability, speed, low cost, and ability to provide answers with efficient and satisfactory interpretation^(7)^. It is important that a protocol for screening purposes be formally elaborated and psychometrically tested so that, for the selection of items, the results of an extensive and rich literature review, empirical experiences of researchers with an idea built for such an instrument are considered, in addition to syntactic and semantic aspects that contribute to clarity, relevance, coherence, and scope of questions to be applied to the population^(7)^.

From this perspective, when tracking voice disorders it is important to measure aspects that contribute to the identification of dysphonia, such as personal factors, occupational risk factors, or vocal manifestations themselves^(5,8)^. A recent study has proposed a screening instrument called Brazilian Dysphonia Screening Tool (Br-DST), prepared from items from the “Voice Handicap Index - VHI” and “Voice Symptoms Scale - VoiSS,” which showed high indexes of sensitivity and diagnostic accuracy using logistic regression models and other statistical measurements such as *Odds Ratio* (OR) and probability estimates for data analysis^(9)^. For use in Brazil, the translation of the title of this instrument into *Instrumento de Rastreio da Disfonia* (IRD^BR^) is important; this nomenclature that will be used throughout this work.

The IRD^BR^ is an instrument composed of only two items with a dichotomous response scale (yes/no), which leads to different decisions based on the responses obtained. These items were selected based on the analysis of statistical relevance of each item of the original instruments. Therefore, the set of questions that presented the greatest association with the presence of the vocal disorder was chosen. That work proved that there are more significant items than others when the aim is to identify the presence of dysphonia and that issues identified as the most relevant may form a quick and simple instrument for its screening^(9)^.

Another screening instrument to identify the risk of dysphonia was previously proposed in the literature with high efficiency in the discrimination of individuals with and without dysphonia in different sample groups^(10)^. This is the instrument called Dysphonia Risk Screening Protocol (*Protocolo de Rastreio do Risco de Disfonia - PRRD*), which consists of 18 questions and uses a 10-cm visual analogue scale, with score calculation for individuals of different age groups with and without vocal complaints. The PRRD is a general protocol designed for gender-independent and professional voice use; it is applicable only to adults and the elderly^(10)^.

However, the aforementioned instrument consists of a structure of 18 questions and a score obtained through the value extracted from a 10-cm visual analogue scale plus an overall score, along with other partial scores. The structure of the instrument is not so simple and, therefore, less viable for use in population-based screening procedures; it is, therefore, a more effective tool for use in traditional individualized procedures in voice clinics.

Also, there are other screening protocols for dysphonia genuinely developed and validated in Brazil. The Screening Index for Voice Disorder - SIVD was developed to identify voice disorders in teachers^(11)^, and the instrument for Screening for Voice Disorders in the Older Adults (*Rastreamento de Alterações Vocais em Idosos* - RAVI)^(12)^ was developed specifically for the elderly population. Both, although they have a proven efficiency in their validation processes, have a limited use to a specific audience and are not indicated for the general population.

Thus, the objective of this study is to compare the efficiency of the *Instrumento de Rastreio da Disfonia* (IRD^BR^), and the traditional self-assessment instruments: Voice-Related Quality of Life (V-RQOL), Voice Handicap Index (VHI), and Voice Symptoms Scale (VoiSS) for dysphonia screening.

## METHODS

This is a quantitative, cross-sectional and retrospective study, evaluated and approved by the Research Ethics Committee of the Institution of origin, with opinion no. 3,470,951/19. As this is a documentary research, the use of the Informed Consent (IC) was waived, and the consent of the laboratory that stores and has responsibility for the data used was required.

Data were extracted from a pre-existing digital database belonging to the voice research laboratory of a higher education institution. This database stores clinical data from patients of both sexes and all age groups who voluntarily sought speech therapy at the speech therapy school clinic linked to this laboratory and presented a voice-related complaint. Individuals aged between 18 and 78 years were included who presented all the information related to vocal anamnesis, auditory-perceptual voice assessment, and laryngological assessment and those who answered all the items of the self-assessment questionnaires selected for this study.

Data from 262 individuals were included; they had a mean age of 41.3 years (SD = 14.5), a minimum of 18 and a maximum of 78 years (maximum age recorded in the database used) and were allocated into two groups: dysphonic (D) and non-dysphonic (ND). Most participants were female, non-voice professionals, and dysphonic. All participants who reported in the anamnesis using their voice as the main work tool were called voice professionals. Regarding the type of dysphonia, there was a higher percentage of behavioral dysphonia. Regarding the intensity of the deviation, mild dysphonia was the majority in relation to moderate and intense dysphonia (Table 1).

**Table 1:** Distribution of participants in relation to age group, gender, professional use of voice, intensity of vocal deviation, and type of dysphonia

Variable		N	%
Gender	Female	197	75.2
	Male	65	24.8
Voice professional	No	165	63.0
	Yes	97	37.0
Intensity of voice deviation	NVQV	25	9.5
	Mild	119	45.4
	Moderate	110	42.0
	Intense	8	3.1
Type of dysphonia in the dysphonic group	Behavioral	220	92.8
	Organic	17	7.2

**Caption:** NVQV = Normal Variability of Quality of Voice

The classification of participants regarding the presence of dysphonia was performed according to the combination of medical and speech-language pathology diagnosis based on laryngeal examination and auditory-perceptual assessment. All dysphonic individuals presented vocal complaints, presence of “structural or functional alteration in the larynx,” and voice quality deviation. The subjects of the ND group did not present vocal complaints. They had a result of “absence of structural or functional alteration of the larynx” recorded in the database and absence of vocal quality deviation.

The variable “vocal deviation intensity” extracted from the research database was obtained through auditory-perceptual analysis of the sustained vowel “é” in maximum phonation time. The analysis was performed using the Vocal Deviation Scale (VDS), a 100-mm visual analogue scale that uses the general degree of deviation (G) to represent the intensity of vocal deviation from the following cut-off points: 35.6 to 50.5 mm mild to moderate deviation; 50.6 to 90.5 mm, moderate deviation; and 90.6 to 100 mm, severe deviation^(13)^. Voices with a score below 35.5 mm were considered to have normal vocal quality variability (NVQV).

The auditory-perceptual analysis of all voices was performed by three speech-language pathologists specialized in voice, with more than ten years of experience in vocal assessment, which contributes to the reliability of the analysis performed. In the assessment session, 20% of the samples were randomly reassessed, and the reliability of the listeners' ratings was analyzed using Cohen's *kappa* coefficient. The results were recorded in the database and accessed to select the voices used in the present study. In this study, only the results of the speech therapist with the highest kappa coefficient (0.80) were used, indicating the judge's good internal reliability.

All responses to the items of the following vocal self-assessment questionnaires, in their translated, adapted and validated versions for Brazilian Portuguese, were also extracted from the database: Voice-Related Quality of Life - V-RQOL, which measures voice-related quality of life^(14)^; the Voice Handicap Index - VHI^(15)^, and its reduced version the Voice Handicap Index-10, which measures the disadvantage that a vocal disorder may bring to the patient's life^(16)^; and the Voice Symptoms Scale - VoiSS^(17)^, which assesses the self-perception of vocal symptoms and the impact produced by the voice disorder.

The V-RQOL has ten items divided into two domains: socio-emotional and physical. It is the only instrument that uses a specific calculation to obtain its total and domain scores. For its interpretation, it is understood that the higher the score, the better the voice-related quality of life^(14)^. The cut-off point established to indicate the presence of dysphonia through the V-RQOL is 91.25 points for the total score^(18)^, with sensitivity indexes of 0.97 and efficiency of 0.91.

The VHI has 30 items divided into three domains: emotional, physical and organic. It has a total score expressed by the simple sum of the responses obtained in all items, which may range from 0 to 120 points^(15)^. Its reduced version, the VHI-10, has ten items. It produces a single total score calculated by the simple sum of the answers to items, which may vary from 0 to 40 points^(16)^. For both instruments, the higher the score produced, the worse the disadvantage perceived by the individual. The cutoff points established to indicate the presence of dysphonia are 19 points for the original version's total score, with maximum indexes of sensitivity and efficiency (=1.00), and 7.5 points for the short version, with sensitivity index of 0.98 and efficiency index of 0.99^(18)^.

The VoiSS also has 30 items divided into three domains: limitation, emotional and physical. It is currently considered the most rigorous and psychometrically more robust instrument for vocal self-assessment^(17)^. The VoiSS allows obtaining data on functionality, emotional impact, and physical symptoms that a voice problem may trigger in an individual's life. The total score is obtained through the simple sum of answers, which can range from 0 to 120. The higher the score, the greater the perception of the general level of vocal alteration in relation to limitations in voice use, emotional reactions, and physical symptoms by the patient. The cut-off point established to indicate the presence of dysphonia through the VoiSS is 16 points, with maximum sensitivity and efficiency indexes (=1.00)^(18)^.

The dysphonia screening tool called “*Instrumento de Rastreio da Dysphonia* (IRD^BR^)” has only two questions and was created from the analysis of items of the three instruments mentioned above: V-RQOL, VHI, and VoiSS. The use of logistic regression models and other statistical decision-making techniques to analyze these traditional instruments results in a new two-item structure with high levels of sensitivity and diagnostic accuracy for the identification of dysphonia. The objective is to track individuals easily and quickly with a high probability of having any vocal disorder in order to properly select and refer those who need a diagnostic evaluation and other specialized procedures^(9)^.

The IRD^BR^ is composed of two questions with dichotomous answers (yes/no): 1) “Do I feel like I have to force my voice for it to come out?” and 2) “Is my voice hoarse?” There are three decision rules guided by the instrument, which are based on the answers of the individual (Annex A). An answer “yes” to both items indicates a high probability of dysphonia and guides immediate referral for detailed diagnostic evaluation (Decision A); an answer “yes” only the for item 2 indicates a moderate probability of dysphonia and recommends a personalized vocal guidance and the need for monitoring voice (Decision B); finally, any other type of answer (“no” to both items or “yes” only to item 1) indicates a low probability of dysphonia and recommends personalized vocal guidance without the immediate need for referral to complementary assessments (Decision C). The instrument has a sensitivity index of 0.86 and an efficiency index of 0.83 for the decision recommended^(9)^.

To analyze the assertiveness of the instruments in relation to the vocal diagnosis of individuals, the cutoff points established for V-RQOL, VHI, and VoiSS^(18)^ and the decision rules A and B recommended by the IRD^BR^ were considered. They point to a high and moderate probability of dysphonia (answer “yes” to both items or only item 2)^(9)^. The aim is to compare the efficiency of IRD^BR^ and traditional instruments of vocal self-assessment that originated it.

Descriptive analysis of variables was performed using mean, standard deviation, and frequency distribution with the aim of characterizing the sample. The Kolmogorov Smirnov normality test was used to confirm the hypothesis of non-normality of data and guide the use of non-parametric hypothesis tests (α= 0.05). Exploratory data analysis was performed using the Mann-Whitney test and the Pearson Chi-square test to compare mean scores of instruments with each other and verify associations between the distribution of data and the variables studied. Statistical analysis was performed using the software R, version 3.5.1, and SPSS, version 23.0. The significance level was 0.05 for all results.

## RESULTS

The comparison of means of total scores in self-assessment instruments for dysphonic (D) and non-dysphonic (ND) groups showed that all instruments have different means for both groups, which indicates that their scores adequately discriminate vocal disorders. However, the non-dysphonic group has a mean value above the cut-off point for normal individuals in all instruments (Table 2).

**Table 2:** Comparison of means of total scores of the self-assessment instruments V-RQOL, VHI, and VoiSS in the dysphonic (D) and non-dysphonic (ND) groups

Instrument	D		ND		p-value
	**Mean**	**SD**	**Mean**	**SD**	
V-RQOL	68.3	20.4	78.8	18.8	0.008*
VHI	49.3	31.0	27.2	23.4	<0.001*
VHI-10	17.0	10.6	9.4	8.1	0.001*
VoiSS	52.0	24.6	34.6	21.4	0.001*

* Significant values at the level α = 0.05

Mann Whitney test

**Caption:** D = Dysphonic; ND = Non-dysphonic; SD = Standard Deviation; V-RQOL = Voice-Related Quality of Life; VHI = Voice Handicap Index; VoiSS = Voice Symptom Scale

The means of total scores of the instruments V-RQOL, VHI, VHI-10, and VoiSS were compared between groups in terms of gender, professional voice use, and type of dysphonia (Table 3). There was a difference only in relation to VoiSS scores for the variable gender, suggesting that females have a higher participation in the total score of this instrument than males do. There were no differences in the other instruments. There were also no differences between the type of dysphonia and the professional use of voice or not regarding the scores of all instruments, which suggests that they are sensitive to capture the impacts of dysphonia in a similar way in both voice professionals and non-professionals and all types of dysphonia (Table 3).

**Table 3:** Comparison of means of total scores of the self-assessing instruments V-RQOL, VHI, VHI-10, and VoiSS between groups in terms of gender, professional voice use, and type of dysphonia

Instrument	Variable		Mean	SD	p-value
V-RQOL	Gender	Female	68.3	20.0	0.082
		Male	72.3	21.9	
	Professional use of voice	No	69.2	20.6	0.907
		Yes	69.4	20.6	
	Type of dysphonia	Behavioral	68.7	20.4	0.304
		Organic	63.4	21.0	
VHI	Gender	Female	48.4	30.1	0.152
		Male	43.6	33.5	
	Professional use of voice	No	49.5	31.6	0.138
		Yes	43.5	29.6	
	Type of dysphonia	Behavioral	49.0	30.8	0.503
		Organic	55.1	33.9	
VHI-10	Gender	Female	16.6	10.4	0.229
		Male	15.0	10.9	
	Professional use of voice	No	16.7	10.8	0.317
		Yes	15.4	10.2	
	Type of dysphonia	Behavioral	16.8	10.5	0.397
		Organic	19.2	11.5	
VoiSS	Gender	Female	52.0	24.0	0.022*
		Male	44.8	26.7	
	Professional use of voice	No	52.0	25.0	0.161
		Yes	47.2	24.3	
	Type of dysphonia	Behavioral	51.7	24.8	0.538
		Organic	54.9	22.2	

* Significant values at the level α = 0.05

Mann Whitney test

**Caption:** V-RQOL = Voice-Related Quality of Life; VHI = Voice Handicap Index; VoiSS = Voice Symptom Scale; SD = Standard Deviation

Finally, an association analysis was performed between the proportion of dysphonic individuals identified by the instruments and the previous diagnosis of dysphonia. At this stage, the cutoff points of V-RQOL, VHI, VHI-10, VoiSS, and the decision rules guided by the IRD^BR^ were used to identify the vocal disorder. The VoiSS presented the highest percentage of correct answers in the identification of the presence of dysphonia, followed by the IRD^BR^ and V-RQOL, both with the same percentage of correct answers (86.1%) and, in sequence, VHI and VHI-10. The IRD^BR^, with only two items, has a high rate of success in identifying dysphonia, which is very close to the first instrument (VoiSS), which has a much higher number of items (Table 4).

**Table 4:** Analysis of the frequency distribution regarding the identification of dysphonia performed by the cutoff point of the instruments V-RQOL, VHI, VoiSS, and IRD^BR^ in relation to the diagnosis of participants

Instrument	Dysphonia classification	D	ND	p-value	
		**n (%)**	**n (%)**		
V-RQOL	Yes	204 (86.1)	17 (68.0)	0.018*	
	No	33 (13.9)	8 (38.0)		
VHI	Yes	192 (81.0)	12 (48.0)	<0.001*	
	No	45 (19.0)	13 (52.0)		
VHI-10	Yes	184 (77.6)	12 (48.0)	0.001*	
	No	53 (22.4)	13 (52.0)		
VoiSS	Yes	220 (92.8)	19 (76.0)	0.005*	
	No	17 (7.2)	6 (24.0)		
IRD^BR^	Yes	204 (86.1)	9 (36.0)	<0.001*	
	No	33 (13.9)	16 (64.0)		
Total		237 (100.0)	25 (100.0)	-	

* Significant values at the level α = 0.05

Chi Square Test

**Caption:** D = Dysphonic; ND = Non-dysphonic; V-RQOL = Voice-Related Quality of Life; VHI = Voice Handicap Index; VoiSS = Voice Symptom Scale; IDR^BR^ = *Instrumento de Rastreio da Disfonia*

In order to observe the general assertiveness of the instruments to classify the presence and absence of dysphonia, the correct answers and errors in the classification of dysphonia of research participants were taken into account. The results showed that the VoiSS ranks first regarding the assertiveness index, followed by the IRD^BR^, which this time performed better than the V-RQOL (Table 5).

**Table 5:** General assertiveness rates of the instruments studied regarding the presence and absence of dysphonia

Instrument	Right n (%)	Wrong n (%)	Total (%)
VoiSS	226 (86.3)	36 (13.7)	**262 (100.0)**
IRD^BR^	220 (84.0)	42 (16.0)	
V-RQOL	212 (80.9)	50 (19.1)	
VHI	205 (78.2)	57 (21.8)	
VHI-10	197 (75.2)	65 (24.8)	

**Caption:** V-RQOL = Voice-Related Quality of Life; VHI = Voice Handicap Index; VoiSS = Voice Symptom Scale; IDR^BR^ = *Instrumento de Rastreio da Disfonia*

## DISCUSSION

Vocal self-assessment has a great relevance in the investigative process of discovering dysphonia, as it is capable of offering information that goes beyond the clinical perspective, informing the impacts of dysphonia according to the patient's own perception. The self-assessment instruments characterize the dysphonia involvement in the physical, social, and emotional dimensions of the dysphonic patient. Thus, it has important contributions to the diagnosis and monitoring of dysphonia cases^(19,20)^.

All self-assessment instruments used in this research are recommended for different samples and are safe to differentiate groups^(14-17)^. In fact, in this research, the scores were determinant to discriminate between the dysphonic group and the non-dysphonic group. However, despite the significant difference found between the means of groups in all instruments, the mean of their scores were higher than their respective cut-off points in the non-dysphonic group.

It is understood that these values may have been influenced by the allocation environment of the participants that are part the research's data, i.e., those attending the voice outpatient clinic of a Speech-Language Pathology Clinic-School of a Higher Education Institution. Even though individuals did not present a diagnosis of dysphonia, the very will to attend service characterizes a perception of some aspect that motivates the desire or need for care, which leads to the modification of the score of the self-assessment instruments. It is also possible to state that these instruments have items that encompass several aspects of the manifestation of dysphonia that often do not have a direct relationship with the clinician's perception^(21)^.

There was no difference regarding the scores of the instruments' scores in relation to the sociodemographic variables analyzed. This indicates that the instruments studied are sensitive to capture the impacts of dysphonia in a similar way for men, women, voice professionals or not, regardless of the type of dysphonia presented. This is confirmed by the literature^(18,22-24)^. Only for the VoiSS there was a difference in relation to gender. The scores were higher for females than for males, a fact that may be related to the higher prevalence of dysphonia and vocal symptoms in women due to the anatomophysiological predisposition that women have to develop voice problems^(22-23,25)^.

As the literature shows, the traditional vocal self-assessment instruments used in this research have cutoff points with a high discriminatory power to differentiate between dysphonic and vocally healthy individuals. They are established based on statistical sensitivity and specificity criteria^(18)^. In this research, all instruments showed high levels of efficacy in that classification, corroborating the reports of previous studies^(17-18)^.

When ranking the instruments in relation to the highest efficiency index, the VoiSS, already considered in the literature as the most psychometrically robust and widely validated vocal self-assessment instrument currently available^(1,17-18,26)^, was more assertive in identifying dysphonia. It is an instrument with a high degree of validity, reliability, and responsiveness regarding vocal changes; it is considered as a perfect classifier in the discrimination of patients with and without vocal disorders^(18,26-27)^. The IRD^BR^ and the V-RQOL are tied in the second ranking in relation to assertiveness rates.

The performance of the IRD^BR^ is remarkable. This recently-developed instrument allows the classification of dysphonia in a shorter and more efficient way, with shorter application time and high discriminative capacity. It proposes a quick, simple and effective investigation of dysphonia, and seems to be characterized as the most viable alternative in screening procedures. Its two items are related to the aspects “hoarseness” and “vocal effort,” which are important symptoms in the investigation of the impact of a possible dysphonia in the individual's life. As it presents a direct correlation with changes in the physiological mechanism of vocal production present in most voice disorders, individuals who have a hoarse and dry voice are approximately three times more likely to be dysphonic^(8,21,26,28)^.

Thus, the results presented by the instrument IRD^BR^ are in line with the literature because, according to the patient's self-report, the sensation of vocal effort and hoarseness are strongly associated with the presence of dysphonia. These items are weighted in a screening instrument^(9)^.

The relationship between V-RQOL indexes and the presence of dysphonia is not consensual in the literature; However, most studies that address this issue point to significant differences in V-RQOL scores between individuals with and without voice disorders^(29-30)^. The high assertiveness index from the cut-off point of this instrument confirms its effectiveness in identifying dysphonic individuals, as already mentioned in a previous study^(18)^.

The VHI and the VHI-10 were the instruments that obtained the lowest percentage of correct answers for the identification of dysphonia and in the analysis of general assertiveness compared to the other instruments studied. However, in the literature, a strong relationship between VHI scores and the patient's vocal diagnosis frequently occurs, and this instrument is thus considered a perfect classifier to identify the presence of dysphonia^(18,31-32)^. Also, the VHI-10 is also considered sensitive to different populations and for the detection of small vocal alterations from the assessment of impacts of a voice problem using a low number of items^(16)^. However, the instrument shortening process was not carried out by factor analysis, and its psychometric criteria were not fully clarified, which weakens its structure. In this study, however, its correctness rates were lower compared to that of other instruments, which leads to the decision not to recommend it in a preferential way in screening actions to detect dysphonia.

Thus, it is possible to state that the IRD^BR^ is a differential tool for the detection of dysphonia in screening procedures considering its short, simple, easy, and quick structure associated with its high levels of efficiency in comparison to instruments used as a reference in vocal self-assessment. The advantages of this instrument and, above all, its feasibility of application in collective actions involving population groups characterize it as the best option for the screening of voice disorders. However, it should be noted that this tool should be used exclusively for screening purposes and that, under no circumstances, replaces the complete speech-language pathology and otorhinolaryngological assessment.

As a limitation of this study, the low number of patients with organic dysphonia in the sample composition is highlighted. The balance in the sample quantity in relation to the different types of dysphonia must be explored in order to ensure that the effectiveness of the instrument is more strongly proven. Another limitation is the use of auditory-perceptual analysis by only one of the three available judges and the absence of inter-evaluator reliability analysis. Despite the extensive previous experience and the high level of internal reliability of the selected judge, the inter-evaluator analysis could bring more robustness to the choice of a single analysis for classifying the study participants.

## CONCLUSION

Vocal self-assessment instruments are highly efficient tools for screening voice disorders in population groups. Among them, the VoiSS has the highest assertiveness index in the identification of dysphonia, followed by the IRD^BR^, instruments that can be considered the most suitable for this procedure. The IRD^BR^ is a recent tool, short, simple, and easy to apply by any health professional. It has a high efficiency for dysphonia tracking.

## Annex A. Answer the two items below, considering your current voice:

**Table:**

**INSTRUMENTO DE RASTREIO DA DISFONIA - IRD^BR^** ***BRAZILIAN DYSPHONIA SCREENING TOOL (Br-DST)*** (Oliveira et al., 2023^9^)		
Question	Answer	Odds ratio for dysphonia (Yes/No)
1) I feel as though I have to strain to produce voice?	( ) Yes ( ) No	2.6
2) My voice is hoarse	( ) Yes ( ) No	11.4
**DECISION GUIDELINES** **(Sensitivity = 87.5%, Specificity = 68.6%, and Accuracy = 83.4%)**		
A) Answer “yes” to both questions →	Probability of dysphonia of 89.2% →	Referral for detailed vocal assessment
B) Answer “yes” to question 2 →	Probability of dysphonia of 68.6% →	Vocal guidance + patient monitoring
C) Other results →	Probability of dysphonia below 68.6% →	Vocal guidelines
