# ERRATUM: Comparison of efficiency of different self-assessment instruments for screening dysphonia

**DOI:** 10.1590/2317-1782/20232023062en

**Published:** 2023-06-23

Due to author's honest mistake the article “Comparison of efficiency of different self-assessment instruments for screening dysphonia” (DOI https://doi.org/10.1590/2317-1782/20232021123en) published in CoDAS 2023;35(2):e20210123, was published with an error on page 9.

In Annex A, where the text reads:

**Annex A**. Answer the two items below, considering your current voice:

It should read:

**Annex A**. Instrumento de Rastreio da Disfonia - IRD^BR^

The authors apologize for the errors.

