# Supplementary material for: Comparison of efficiency of different self-assessment instruments for screening dysphonia
Source: Codas. 2023 Apr 17;35(2):e20210123. doi: 10.1590/2317-1782/20232021123en (PMC10145995; doi:10.1590/2317-1782/20232021123en)
Supplement: Translation

# Translation: Comparação da eficiência de diferentes instrumentos de autoavaliação para o rastreio da disfonia

**DOI:** 10.1590/2317-1782/20232021123pt

**Authors:** Priscila Oliveira, Héryka Maria Oliveira Lima, Maiara dos Santos Sousa, Larissa Nadjara Almeida, Hêmmylly Farias da Silva, Ana Celiane Ugulino, Anna Alice Almeida, Leonardo Lopes

**Affiliations:** 1 Departamento de Fonoaudiologia, Centro de Ciências da Saúde, Universidade Federal da Paraíba - UFPB - João Pessoa (PB), Brasil.; 2 Departamento de Estatística, Universidade Federal da Paraíba - UFPB - João Pessoa (PB), Brasil.

**Keywords:** Voz, Disfonia, Autoteste, Programas de Rastreamento, Tomada de Decisões, Fonoaudiologia

## Abstract

**Objetivo:**

Comparar a eficiência de diferentes instrumentos de autoavaliação vocal para o rastreio da disfonia.

**Método:**

Participaram 262 indivíduos disfônicos e não disfônicos, com média de idade de 41,3 (±14,5) anos. O diagnóstico da disfonia foi dado a partir da análise perceptivo-auditiva da vogal sustentada “é” e do diagnóstico laringológico. Foram coletadas as respostas dos instrumentos: Questionário de Qualidade de Vida em Voz (QVV), Índice de Desvantagem Vocal (IDV), IDV-10, Escala de Sintomas Vocais (ESV) e do Br-DST (*Brazilian Dysphonia Screening Tool*), denominado no português brasileiro como Instrumento de Rastreio da Disfonia (IRD^BR^). Para análise da assertividade destes em relação à presença da disfonia, foram utilizados os pontos de corte de cada instrumento e a regra de decisão preconizada pelo IRD^BR^. Foi realizada uma análise exploratória para comparação das médias dos escores dos instrumentos e verificação de associações entre as variáveis.

**Resultados:**

Os instrumentos avaliados foram sensíveis para captar o impacto da disfonia de forma semelhante independentemente do uso profissional da voz e tipo de disfonia. Foi observada diferença apenas nos escores da ESV para a variável sexo, com maior pontuação observada no sexo feminino. Em relação à assertividade global, os instrumentos apresentaram elevados índices de acerto na classificação, com destaque para a ESV que apresentou maior índice (86,3%), seguida do IRD^BR^ (84,0%), QVV (80,9%), IDV (78,2%) e IDV-10 (75,2%).

**Conclusão:**

A ESV apresenta maior índice de assertividade na identificação da disfonia, seguida do IRD^BR^. O IRD^BR^ é uma ferramenta curta, simples e de fácil aplicação para procedimentos de rastreio.

## INTRODUÇÃO

Os distúrbios de voz acometem de 3 a 9% da população e produzem uma série de impactos negativos na qualidade de vida dos indivíduos^(1,2)^. Por isso, destaca-se a importância de uma avaliação vocal completa e eficiente para o diagnóstico de um possível distúrbio de voz, que contemple análises perceptivo auditiva, acústica e aerodinâmica da voz, além do exame laríngeo e da avaliação na perspectiva do paciente^(3)^.

No entanto, a realização de um procedimento completo de avaliação vocal em larga escala nem sempre é viável, pois o diagnóstico de um distúrbio de voz é um processo que demanda tempo, recursos materiais e profissionais especializados para sua realização. Em inquéritos populacionais ou campanhas preventivas, mecanismos específicos para rastreio são mais recomendados, pois visam selecionar previamente indivíduos com elevadas chances de apresentar a disfonia a fim de serem encaminhados para uma avaliação diagnóstica completa confirmatória em um momento posterior^(4)^. Sendo assim, uma ferramenta de rastreio não possui a necessidade de ser um instrumento completo de diagnóstico. A utilização de ferramentas curtas de rastreio para a pré-seleção de indivíduos de risco a serem encaminhados para confirmação diagnóstica posterior pode permitir a ampliação do alcance e da resolutividade das ações epidemiológicas preventivas na área de Voz^(5,6)^.

Os requisitos indispensáveis para um instrumento de rastreio são: fácil aplicação, ampla usabilidade, rapidez, de baixo custo e que forneçam respostas com interpretação eficiente e satisfatória^(7)^. É importante que um protocolo com fins de triagem seja formalmente elaborado e psicometricamente testado de modo que, para a seleção dos seus itens, sejam considerados os resultados de uma extensa e rica revisão de literatura, experiências empíricas dos pesquisadores com a ideia construída para tal instrumento, além de aspectos sintáticos e semânticos que contribuam para a clareza, pertinência, coerência e abrangência das questões a serem aplicadas para a população^(7)^.

Nessa perspectiva, no rastreamento de distúrbios vocais, é importante que sejam mensurados aspectos que contribuam para a identificação de uma disfonia, como fatores pessoais, fatores de riscos ocupacionais ou manifestações vocais propriamente ditas^(5,8)^. Um estudo recente apresentou como proposta um instrumento de rastreio denominado *Brazilian Dysphonia Screening Tool* (Br-DST), elaborado a partir de itens dos questionários “Índice de Desvantagem Vocal - IDV” e “Escala de Sintomas Vocais - ESV”, que demonstrou elevados índices de sensibilidade e acurácia diagnóstica utilizando modelos de regressão logística e outras medidas estatísticas como *Odds Ratio* (OR) e estimativas de probabilidade para análise de dados^(9)^. Para utilização no Brasil, sugere-se a tradução do título desse instrumento para Instrumento de Rastreio da Disfonia (IRD^BR^), nomenclatura que será utilizada no decorrer desse trabalho.

O IRD^BR^ é um instrumento composto por apenas dois itens com escala de resposta dicotômica (sim/não), que direcionam para decisões diferentes a partir das respostas obtidas. Esses itens foram selecionados a partir da análise da relevância estatística de cada um dos itens dos instrumentos originais, sendo escolhidos, portanto, o conjunto de questões que apresentou maior associação com a presença do distúrbio vocal. O trabalho comprovou que existem itens mais significativos em relação a outros quando se almeja identificar a presença da disfonia e que esses identificados como mais relevantes podem compor um instrumento rápido e simples para o seu rastreio^(9)^.

Um outro instrumento de triagem para identificar o risco de disfonia foi anteriormente proposto na literatura com elevada eficiência na discriminação de indivíduos com e sem disfonia em diferentes grupos amostrais^(10)^. Trata-se do instrumento intitulado como *Dysphonia Risk Screening Protocol* ou Protocolo de Rastreio do Risco de Disfonia - PRRD, que é composto por 18 questões e utiliza uma escala visual analógica de 10 cm, com cálculo de pontuação para indivíduos de diferentes faixas etárias com e sem queixas vocais. O PRRD é um protocolo geral projetado para uso independente de sexo e uso profissional da voz, aplicável apenas para adultos e idosos^(10)^.

No entanto, o referido instrumento consiste em uma estrutura de 18 questões e um escore obtido por meio do valor extraído de uma escala visual analógica de 10 cm adicionado a uma pontuação geral, juntamente com outras pontuações parciais. Considera-se que a estrutura do instrumento não é tão simples e, por isso, menos viável para utilização em procedimentos populacionais de triagem sendo, portanto, uma ferramenta mais eficaz para uso em procedimentos tradicionais individualizados nas clínicas de voz.

Além deste, existem outros protocolos de rastreio para disfonia genuinamente desenvolvidos e validados no Brasil. O Índice de Triagem para Distúrbio de Voz - ITDV foi desenvolvido para identificação de distúrbios vocais em professores^(11)^, e o instrumento de Rastreio de Alterações Vocais no Idoso - RAVI^(12)^ foi desenvolvido especificamente para a população idosa. Ambos, embora apresentem eficiência comprovada em seu processo de validação, têm uso limitado a um público específico e não são indicados para a população em geral.

Assim, o objetivo desse estudo foi comparar a eficiência do Instrumento de Rastreio da Disfonia (IRD^BR^), e dos instrumentos de autoavaliação tradicionais: Questionário de Qualidade de Vida em Voz (QVV), Índice de Desvantagem Vocal (IDV) e Escala de Sintomas Vocais (ESV), para o rastreio da disfonia.

## MÉTODO

Esta é uma pesquisa quantitativa, transversal e retrospectiva, avaliada e aprovada pelo Comitê de Ética em Pesquisa da Instituição de origem, com o parecer número 3.470.951/19. Por se tratar de uma pesquisa documental, o uso de Termo de Consentimento Livre e Esclarecido (TCLE) foi dispensado e a anuência do laboratório que armazena e tem a responsabilidade sobre os dados utilizados foi requerida.

Os dados foram extraídos de um banco de dados digital pré-existente pertencente ao laboratório de pesquisa em Voz de uma instituição de ensino superior. Tal banco de dados armazena dados clínicos de pacientes de ambos os sexos e todas as faixas etárias, que procuraram voluntariamente o atendimento fonoaudiológico na clínica-escola de Fonoaudiologia vinculada a esse laboratório, apresentando alguma queixa relativa à voz. Foram incluídos indivíduos com idade entre 18 e 78 anos, que apresentaram todas as informações relativas à anamnese vocal, avaliação perceptivo-auditiva da voz e avaliação laringológica, e que responderam a todos os itens dos questionários de autoavaliação selecionados para este estudo.

Foram incluídos dados de 262 indivíduos, com média de idade de 41,3 anos (DP = 14,5), mínima de 18 e máxima de 78 anos (idade máxima registrada no banco de dados utilizado), que foram alocados em dois grupos: disfônicos (D) e não disfônicos (ND). A maioria dos participantes foram do sexo feminino, não profissionais da voz e disfônicos. Foram denominados como profissionais da voz todos os participantes que referiram, na anamnese, utilizar a voz como principal ferramenta de trabalho. Em relação ao tipo de disfonia, observou-se um percentual maior de disfonias comportamentais e quanto à intensidade do desvio, disfonias de grau leve foram a maioria em relação a disfonias de grau moderado e intenso (Tabela 1).

**Tabela 1:** Distribuição dos participantes em relação à faixa etária, sexo, uso profissional da voz, intensidade do desvio vocal e tipo da disfonia

Variável		N	%
Sexo	Feminino	197	75,2
	Masculino	65	24,8
Profissional da voz	Não	165	63,0
	Sim	97	37,0
Intensidade do desvio vocal	VNQV	25	9,5
	Leve	119	45,4
	Moderada	110	42,0
	Intensa	8	3,1
Tipo de disfonia no grupo disfônico	Comportamental	220	92,8
	Orgânica	17	7,2

**Legenda:** VNQV = Variabilidade Normal da Qualidade Vocal

A classificação dos participantes quanto à presença de disfonia foi realizada de acordo com a combinação do diagnóstico médico e fonoaudiológico, a partir do exame laríngeo e da avaliação perceptivo-auditiva. Todos os indivíduos disfônicos apresentaram queixa vocal, presença de “alteração estrutural ou funcional na laringe” e desvio da qualidade vocal. Os sujeitos do grupo ND não apresentavam queixa vocal, apresentaram resultado de “ausência de alteração estrutural ou funcional da laringe” registrado na base de dados e ausência de desvio da qualidade vocal.

A variável “intensidade do desvio vocal” extraída do banco de dados da pesquisa, foi obtida por meio de análise perceptivo-auditiva da vogal sustentada “é” em tempo máximo de fonação. A análise foi realizada por meio da Escala de Desvio Vocal (EDV), uma escala visual analógica de 100 mm que utiliza o grau geral de desvio (G) para representar a intensidade do desvio vocal, a partir dos seguintes pontos de corte: 35,6 a 50,5 mm desvio leve a moderado, 50,6 a 90,5 mm desvio moderado e 90,6 a 100 mm desvio intenso^(13)^. As vozes com pontuação inferior até 35,5 mm foram consideradas com variabilidade normal da qualidade vocal (VNQV).

A análise perceptivo-auditiva de todas as vozes foi feita por três fonoaudiólogos especialistas em voz com mais de 10 anos de experiência em avaliação vocal, o que contribui a favor da confiabilidade da análise realizada. Na sessão de avaliação, 20% das amostras foram reavaliadas aleatoriamente e a confiabilidade das avaliações dos ouvintes foi analisada usando o coeficiente *kappa* de Cohen. Os resultados foram registrados no banco de dados e acessados para a seleção das vozes utilizadas no presente estudo. Nesse estudo, foram utilizados apenas os resultados do fonoaudiólogo com maior coeficiente kappa (0,80), indicando boa confiabilidade interna do juiz.

Também foram extraídas do banco de dados todas as respostas dos itens dos seguintes questionários de autoavaliação vocal, em suas versões traduzidas, adaptadas e validadas para o português brasileiro: Questionário de Qualidade de Vida em Voz - QVV, que mensura a qualidade de vida relacionada à voz^(14)^; o Índice de Desvantagem Vocal - IDV^(15)^, e sua versão reduzida o Índice de Desvantagem Vocal-10, que mensuram a desvantagem que um distúrbio vocal pode trazer para a vida do paciente^(16)^; e a Escala de Sintomas Vocais - ESV^(17)^, que avalia a autopercepção dos sintomas vocais e o impacto produzido pelo distúrbio de voz.

O QVV possui 10 itens divididos em 2 domínios, socioemocional e físico. É o único instrumento que utiliza um cálculo específico para obtenção dos seus escores total e por domínios, e para sua interpretação, entende-se que quanto maior o escore obtido, melhor é a qualidade de vida relacionada à voz^(14)^. O ponto de corte estabelecido para indicar a presença da disfonia por meio do QVV é de 91,25 pontos para o escore total^(18)^, com índices de sensibilidade de 0,97 e eficiência de 0,91.

O IDV possui 30 itens divididos em 3 domínios, emocional, físico e orgânico, e possui escore total expresso pelo somatório simples das respostas obtidas em todos os itens, que pode variar de 0 a 120 pontos^(15)^. Já sua versão reduzida o IDV-10, possui 10 itens, e produz um escore total único, calculado pelo somatório simples das respostas de seus itens, que pode variar de 0 a 40 pontos^(16)^. Para ambos instrumentos, quanto maior o escore produzido, pior é a desvantagem percebida pelo indivíduo. Os pontos de corte estabelecidos para indicar a presença da disfonia são de 19 pontos para o escore total da versão original, com índices máximos de sensibilidade e eficiência (=1,00), e de 7,5 pontos para a versão reduzida, com índices de sensibilidade de 0,98 e eficiência de 0,99^(18)^.

A ESV também possui 30 itens divididos em 3 domínios, limitação, emocional e físico, e atualmente é considerada o instrumento mais rigoroso e psicometricamente mais robusto para a autoavaliação vocal^(17)^. Por meio da ESV é possível obter dados sobre funcionalidade, impacto emocional e sintomas físicos que um problema de voz pode desencadear na vida de um indivíduo. O escore total é obtido por meio de somatório simples das respostas, que pode variar de 0 a 120, e quanto maior o escore maior é a percepção do nível geral de alteração vocal em relação à limitação no uso da voz, reações emocionais e sintomas físicos por parte do paciente. O ponto de corte estabelecido para indicar a presença da disfonia a partir do escore total é de 16 pontos, com índices máximos de sensibilidade e eficiência (=1,00)^(18)^.

Já a ferramenta para rastreio da disfonia denominada “Instrumento de Rastreio da Disfonia (IRD^BR^)” possui apenas duas questões a serem investigadas e foi elaborada a partir da análise dos itens dos três instrumentos referidos anteriormente: QVV, IDV e ESV. A utilização de modelos de regressão logística e outras técnicas estatísticas de tomada de decisão na análise desses instrumentos tradicionais resultaram em uma nova estrutura de dois 2 itens com elevados índices de sensibilidade e acurácia diagnóstica para identificação da disfonia. Seu objetivo é rastrear, de forma simples e rápida, indivíduos com alta probabilidade de apresentarem qualquer distúrbio vocal, a fim de selecionar e encaminhar adequadamente aqueles que precisem de uma avaliação diagnóstica e demais condutas especializadas^(9)^.

O IRD^BR^ é composto por duas perguntas com respostas dicotômicas (sim/não): 1) “Sinto que tenho que fazer força para minha voz sair?” e 2) “Minha voz é rouca?”. São três regras de decisão direcionadas pelo instrumento, que se baseiam nas respostas apresentadas pelo indivíduo (Anexo A). A resposta “sim” aos dois itens indica elevada probabilidade de disfonia e orienta o encaminhamento imediato para avaliação diagnóstica detalhada (Decisão A); a resposta “sim” apenas para o item 2 indica uma probabilidade moderada de disfonia e orienta o oferecimento de orientações vocais personalizadas e a necessidade de monitoramento do caso (Decisão B); por fim, qualquer outro tipo de resposta (“não” para os dois itens ou “sim” apenas para o item 1) indica baixa probabilidade de disfonia, e direciona o oferecimento de orientações vocais personalizadas, sem necessidade imediata de encaminhamento para avaliações complementares (Decisão C). O instrumento apresenta com índices de sensibilidade de 0,86 e eficiência de 0,83 para a decisão preconizada^(9)^.

Para analisar a assertividade dos instrumentos em relação ao diagnóstico vocal dos indivíduos, foram utilizados os pontos de corte estabelecidos para o QVV, IDV e ESV^(18)^ e as regras de decisão A e B preconizadas pelo IRD^BR^, que apontam alta e moderada probabilidade de disfonia (resposta “sim” aos dois itens ou apenas ao item 2)^(9)^. O intuito foi comparar a eficiência do IRD^BR^ e dos instrumentos tradicionais de autoavaliação vocal que o originaram, para a detecção da disfonia.

A análise descritiva das variáveis foi realizada por meio de média, desvio-padrão e distribuição de frequência, com o objetivo de caracterizar a amostra. O teste de normalidade *Komolgorov Smirnov* foi utilizado para confirmar a hipótese de não normalidade dos dados e orientar a utilização de testes de hipótese não paramétricos (α= 0,05). A análise exploratória dos dados foi realizada por meio do teste de *Mann-Whitney* e do teste *Qui-quadrado de Pearson*, para comparar as médias dos escores dos instrumentos entre si e verificar associações entre a distribuição dos dados e as variáveis estudadas. A análise estatística foi realizada por meio dos *softwares* R versão 3.5.1 e SPSS versão 23.0. Considerou-se o nível de significância de 0,05 para todos os resultados.

## RESULTADOS

A comparação das médias dos escores totais dos instrumentos de autoavaliação, dos grupos de disfônicos (D) e não disfônicos (ND), mostrou que todos os instrumentos apresentam médias diferentes para os dois grupos, o que indica que seus escores discriminam adequadamente o distúrbio vocal. No entanto, destaca-se que o grupo não disfônico apresenta média superior ao ponto de corte para indivíduos normais em todos os instrumentos (Tabela 2).

**Tabela 2:** Comparação das médias dos escores totais dos instrumentos de autoavaliação QVV, IDV e ESV nos grupos de disfônicos (D) e não disfônicos (ND)

Instrumento	D		ND		p-valor
	**Média**	**DP**	**Média**	**DP**	
QVV	68,3	20,4	78,8	18,8	0,008*
IDV	49,3	31,0	27,2	23,4	<0,001*
IDV-10	17,0	10,6	9,4	8,1	0,001*
ESV	52,0	24,6	34,6	21,4	0,001*

* Valores significativos ao nível α = 0,05

Teste de Mann-Whitney

**Legenda:** D = Disfônico; ND = Não-disfônico; DP = Desvio-padrão; QVV = Questionário de Qualidade de Vida em Voz; IDV = Índice de Desvantagem Vocal; ESV = Escala de Sintomas Vocais

A comparação das médias dos escores totais dos instrumentos QVV, IDV, IDV-10 e ESV entre grupos com relação ao sexo, uso profissional da voz e tipo de disfonia também foi investigada (Tabela 3). Foi observada diferença apenas em relação aos escores da ESV para a variável sexo, sugerindo que o sexo feminino apresentou maior pontuação para o escore total desse instrumento do que o sexo masculino. Não foram observadas diferenças nos demais instrumentos. Também não foram observadas diferenças entre o tipo de disfonia e o uso profissional da voz ou não quanto aos escores de todos os instrumentos, o que sugere que estes são sensíveis para captar o impacto da disfonia de forma semelhante em profissionais ou não profissionais da voz e em todos os tipos de disfonias (Tabela 3).

**Tabela 3:** Comparação das médias dos escores totais dos instrumentos de autoavaliação QVV, IDV e ESV entre grupos com relação ao sexo, uso profissional da voz e tipo de disfonia

Instrumento	Variáveis		Média	DP	p-valor
QVV	Sexo	Feminino	68,3	20,0	0,082
		Masculino	72,3	21,9	
	Uso profissional da voz	Não	69,2	20,6	0,907
		Sim	69,4	20,6	
	Tipo de disfonia	Comportamental	68,7	20,4	0,304
		Orgânica	63,4	21,0	
IDV	Sexo	Feminino	48,4	30,1	0,152
		Masculino	43,6	33,5	
	Uso profissional da voz	Não	49,5	31,6	0,138
		Sim	43,5	29,6	
	Tipo de disfonia	Comportamental	49,0	30,8	0,503
		Orgânica	55,1	33,9	
IDV-10	Sexo	Feminino	16,6	10,4	0,229
		Masculino	15,0	10,9	
	Uso profissional da voz	Não	16,7	10,8	0,317
		Sim	15,4	10,2	
	Tipo de disfonia	Comportamental	16,8	10,5	0,397
		Orgânica	19,2	11,5	
ESV	Sexo	Feminino	52,0	24,0	0,022*
		Masculino	44,8	26,7	
	Uso profissional da voz	Não	52,0	25,0	0,161
		Sim	47,2	24,3	
	Tipo de disfonia	Comportamental	51,7	24,8	0,538
		Orgânica	54,9	22,2	

* Valores significativos ao nível α = 0,05

Teste de Mann-Whitney

**Legenda:** QVV = Questionário de Qualidade de Vida em Voz; IDV = Índice de Desvantagem Vocal; ESV = Escala de Sintomas Vocais; DP = Desvio-padrão

Por fim, foi realizada a análise de associação entre a proporção de disfônicos identificada pelos instrumentos e pelo diagnóstico prévio da disfonia. Nessa etapa, foram utilizados os pontos de corte do QVV, IDV, IDV-10, ESV e as regras de decisão orientadas pelo IRD^BR^ para identificação do distúrbio vocal. A ESV foi o instrumento que apresentou o maior percentual de acerto na identificação da presença da disfonia, seguida do IRD^BR^ e do QVV, ambos com o mesmo percentual de acerto (86,1%) e, na sequência, IDV e IDV-10. Destaca-se que o IRD^BR^, com apenas 2 itens, apresenta uma elevada taxa de acerto na identificação da disfonia, muito próxima ao primeiro instrumento (ESV) que possui uma quantidade bem superior de itens (Tabela 4).

**Tabela 4:** Análise da distribuição de frequência acerca da identificação da disfonia realizada pelo ponto de corte dos instrumentos QVV, IDV, ESV e IRD^BR^ em relação ao diagnóstico dos participantes

Instrumento	Classificação da disfonia	D	ND	p-valor
		**n (%)**	**n (%)**	
QVV	Sim	204 (86,1)	17 (68,0)	0,018*
	Não	33 (13,9)	8 (38,0)	
IDV	Sim	192 (81,0)	12 (48,0)	<0,001*
	Não	45 (19,0)	13 (52,0)	
IDV 10	Sim	184 (77,6)	12 (48,0)	0,001*
	Não	53 (22,4)	13 (52,0)	
ESV	Sim	220 (92,8)	19 (76,0)	0,005*
	Não	17 (7,2)	6 (24,0)	
IRD^BR^	Sim	204 (86,1)	9 (36,0)	<0,001*
	Não	33 (13,9)	16 (64,0)	
Total		237 (100,0)	25 (100,0)	-

* Valores significativos ao nível α = 0,05

Teste Qui-quadrado

**Legenda:** D = Disfônico; ND = Não-disfônico; QVV = Questionário de Qualidade de Vida em Voz; IDV = Índice de Desvantagem Vocal; ESV = Escala de Sintomas Vocais; IDR^BR^ = Instrumento de Rastreio da Disfonia

Com o intuito de observar a assertividade geral dos instrumentos, tanto para classificar a presença quanto a ausência da disfonia, foram somados os acertos e os erros cometidos por esses na classificação da disfonia dos participantes da pesquisa. Os resultados apontaram que a ESV permanece em primeiro lugar quanto ao índice de assertividade, seguida do IRD^BR^, que dessa vez, apresentou desempenho superior ao QVV (Tabela 5).

**Tabela 5:** Taxas de assertividade geral dos instrumentos estudados quanto à presença e ausência da disfonia

Instrumento	Acertos n (%)	Erros n (%)	Total (%)
ESV	226 (86,3)	36 (13,7)	**262 (100,0)**
IRD^BR^	220 (84,0)	42 (16,0)	
QVV	212 (80,9)	50 (19,1)	
IDV	205 (78,2)	57 (21,8)	
IDV 10	197 (75,2)	65 (24,8)	

**Legenda:** QVV = Questionário de Qualidade de Vida em Voz; IDV = Índice de Desvantagem Vocal; ESV = Escala de Sintomas Vocais; IDR^BR^ = Instrumento de Rastreio da Disfonia

## DISCUSSÃO

A autoavaliação vocal apresenta grande relevância no processo investigativo da manifestação da disfonia, por ser a avaliação capaz de oferecer informações que vão além da perspectiva clínica, informando o impacto da disfonia na percepção do próprio paciente. Os instrumentos de autoavaliação caracterizam o acometimento da disfonia nas dimensões física, social e emocional do paciente disfônico, e assim, trazem importantes contribuições para o diagnóstico e monitoramento dos casos de disfonia^(19,20)^.

Todos os instrumentos de autoavaliação utilizados na pesquisa são recomendados para utilização em diferentes amostras e mostram-se seguros para diferenciar grupos^(14-17)^. De fato, nesta pesquisa, seus escores foram determinantes para discriminar o grupo disfônicos e o grupo não disfônico. No entanto, apesar da diferença significativa encontrada entre as médias dos grupos em todos os instrumentos, foi possível observar que a média dos seus escores estavam superiores aos seus respectivos pontos de corte no grupo não disfônicos.

Compreende-se que esses valores podem ter sido influenciados pelo ambiente de alocação dos participantes que compõem o banco de dados da pesquisa, o ambulatório de Voz de uma Clínica-Escola de Fonoaudiologia de uma Instituição de Ensino Superior. Considera-se que, por mais que os indivíduos não apresentassem diagnóstico de disfonia, a própria procura pelo serviço caracteriza a percepção de algum aspecto que motive o desejo ou a necessidade do atendimento, o que eleva à modificação da pontuação dos instrumentos de autoavaliação. É possível ainda refletir que esses instrumentos possuem itens que englobam diversos aspectos da manifestação da disfonia, que muitas vezes não apresentam relação direta com a percepção do clínico^(21)^.

Não houve diferença quanto à pontuação dos escores dos instrumentos em relação às variáveis sociodemográficas analisadas, indicando que os instrumentos estudados são sensíveis para captar o impacto da disfonia de forma semelhante para homens, mulheres, profissionais da voz ou não, e independentemente do tipo de disfonia apresentado o que é confirmado pela literatura^(18,22-24)^. Apenas para a ESV observou-se diferença em relação ao sexo, com escores maiores para o sexo feminino em relação ao masculino, fato que pode estar relacionado à maior prevalência da disfonia e de sintomas vocais em mulheres, em virtude da predisposição anatomofisiológica que lhe é própria para desenvolver problemas de voz^(22-23,25)^.

Conforme conhecido na literatura, os instrumentos de autoavaliação vocal tradicionais utilizados nesta pesquisa apresentam pontos de corte com elevado poder discriminatório para diferenciar indivíduos disfônicos e vocalmente saudáveis, estabelecidos a partir de critérios de sensibilidade e especificidade estatísticos^(18)^. Nessa pesquisa, observou-se que todos os instrumentos apresentaram elevados índices de eficácia nessa classificação, corroborando o descrito em estudos anteriores^(17-18)^.

Ao ranquear os instrumentos em relação ao maior índice de eficiência, a ESV, já considerado na literatura como o instrumento de autoavaliação vocal mais robusto psicometricamente e amplamente validado que se tem disponível atualmente^(1,17-18,26)^, apresentou-se como mais assertivo em relação à identificação da disfonia. Trata-se de um instrumento com alto grau de validade, confiabilidade e capacidade de resposta quanto a mudanças vocais, considerado como classificador perfeito na discriminação de pacientes com e sem distúrbios vocais^(18,26-27)^. Já o IRD^BR^ e o QVV estiveram empatados em segundo lugar em relação à taxa de assertividade.

O desempenho apresentado pelo IRD^BR^ é destacável. Esse instrumento recentemente desenvolvido permite a classificação da disfonia de forma mais curta e eficiente, com menor tempo de aplicação e elevada capacidade discriminativa. Propõe a investigação rápida, simples e eficaz da disfonia, e parece caracterizar-se como a alternativa mais viável em procedimentos de rastreio. Seus dois itens relacionam-se aos aspectos “rouquidão” e “esforço vocal”, sintomas importantes na investigação do impacto de uma possível disfonia na vida do indivíduo. Por apresentar correlação direta com alterações do mecanismo fisiológico de produção vocal presente na maior parte dos transtornos vocais, indivíduos que possuem a voz rouca e seca têm aproximadamente 3 vezes mais chance de serem disfônicos^(8,21,26,28)^.

Dessa forma, os resultados apresentados pelo instrumento IRD^BR^ encontram-se em consonância com a literatura pois, de acordo com o autorrelato do paciente, a sensação de esforço vocal e o sintoma rouquidão são fortemente associados à presença de disfonia, sendo esses itens de maior peso em um instrumento de rastreio^(9)^.

A relação dos índices do QVV com a presença da disfonia não é consensual na literatura, porém, grande parte dos estudos que abordam essa questão apontam diferenças significativas quanto aos escores do QVV entre indivíduos com e sem distúrbios de voz^(29-30)^. O elevado índice de assertividade a partir do ponto de corte desse instrumento confirma sua eficácia para identificar indivíduos disfônicos, já citada em estudo anterior^(18)^.

O IDV e o IDV-10, foram os instrumentos que obtiveram menor percentual de acerto para identificação da disfonia e na análise da assertividade geral, quando comparados aos outros instrumentos estudados. No entanto, na literatura, a forte relação entre os escores do IDV e o diagnóstico vocal do paciente é observada de forma frequente e esse instrumento é considerado como um perfeito classificador para identificar a presença da disfonia^(18,31-32)^. Adicionalmente, o IDV-10 também é considerado sensível a diversas populações e à detecção de pequenas alterações vocais a partir da avaliação do impacto de um problema de voz utilizando-se uma quantidade reduzida de itens^(16)^. Porém, o processo de redução do instrumento não foi realizado por meio de análise fatorial e seus critérios psicométricos não foram totalmente esclarecidos, o que fragiliza sua estrutura. Nesse estudo, contudo, seus índices de acerto foram menores em relação aos demais instrumentos, o que conduz à decisão de não recomendá-lo de forma preferencial em ações de rastreio para detecção da disfonia.

Dessa forma, é possível afirmar que o IRD^BR^ se mostra como ferramenta diferencial para a detecção da disfonia em procedimentos de rastreio, ao considerar sua estrutura curta, simples, de fácil e rápida de aplicação, associada aos seus elevados índices de eficiência em comparação aos instrumentos utilizados como referência na autoavaliação vocal. As vantagens desse instrumento e, sobretudo, sua viabilidade de aplicação em ações coletivas envolvendo grupos populacionais caracteriza-o como melhor opção a ser utilizada no rastreio dos distúrbios de voz. No entanto, cabe ressaltar que essa é uma ferramenta que deve ser utilizada exclusivamente para fins de rastreio e que, em hipótese alguma, substitui a avaliação fonoaudiológica e otorrinolaringológica completa.

Ressalta-se, como limitação desse estudo o número reduzido de pacientes com disfonias orgânicas na composição da amostra. O equilíbrio no quantitativo da amostra em relação aos variados tipos de disfonias deve ser explorado, a fim de garantir que a eficácia do instrumento seja mais fortemente comprovada. Considera-se como outra limitação a utilização da análise perceptivo-auditiva de apenas um dos três juízes disponíveis e a ausência da análise da confiabilidade inter-avaliadores. Apesar da ampla experiência prévia e do elevado índice de confiabilidade interna do juiz selecionado, a análise inter-avaliadores poderia trazer mais segurança à escolha de uma única análise para classificação dos participantes do estudo.

## CONCLUSÃO

Os instrumentos de autoavaliação vocal são ferramentas com elevada eficiência para o rastreio de distúrbios da voz em grupos populacionais. Entre eles, a ESV apresenta maior índice de assertividade na identificação da disfonia, seguida do IRD^BR^, instrumentos que podem ser considerados os mais indicados para esse procedimento. O IRD^BR^ é uma ferramenta recente, curta, simples e de fácil aplicação por qualquer profissional da saúde, que apresenta elevada eficiência para rastreamento da disfonia.

## Anexo A. Responda aos dois itens abaixo, considerando a sua voz atualmente:

**Table:**

INSTRUMENTO DE RASTREIO DA DISFONIA - IRD^BR^ *BRAZILIAN DYSPHONIA SCREENING TOOL (Br-DST)* (Oliveira et al., 2023^9^)		
Pergunta	Resposta	Razão de chances para a disfonia (Sim/Não)
1) Sinto que tenho que fazer força para a minha voz sair	() Sim () Não	2,6
2) Minha voz é rouca	() Sim () Não	11,4
REGRA DE DECISÃO (Sensibilidade = 87,5%, Especificidade = 68,6% e Acurácia = 83,4%)		
**A) Resposta “sim” para as duas perguntas →**	Probabilidade de disfonia de 89,2% →	Encaminhamento para avaliação vocal detalhada
**B) Resposta “sim” para a pergunta 2 →**	Probabilidade de disfonia de 68,6% →	Orientações vocais + monitoramento do paciente
**C) Demais resultados →**	Probabilidade de disfonia abaixo de 68,6% →	Orientações vocais
