# Supplementary material for: ERRATUM: Comparison of efficiency of different self-assessment instruments for screening dysphonia
Source: Codas. 2023 Jun 23;35(3):e20230062. doi: 10.1590/2317-1782/20232023062en (PMC10319442; doi:10.1590/2317-1782/20232023062en)
Supplement: Translation

# Translation: ERRATA: Comparação da eficiência de diferentes instrumentos de autoavaliação para o rastreio da disfonia

**DOI:** 10.1590/2317-1782/20232023062pt

Devido ao erro honesto dos autores o artigo “Comparação da eficiência de diferentes instrumentos de autoavaliação para o rastreio da disfonia” (DOI https://doi.org/10.1590/2317-1782/20232021123pt), publicado em CoDAS 2023;35(2):e20210123, foi publicado com um erro na página 9.

No Anexo A, onde se lia:

**Anexo A**. Responda aos dois itens abaixo, considerando a sua voz atualmente:

Leia-se:

**Anexo A.** Instrumento de Rastreio da Disfonia - IRD^BR^

Os autores pedem desculpas pelos erros.
